# How nanotechnology-enabled concepts could contribute to the prevention, diagnosis and therapy of bacterial infections

**DOI:** 10.1186/s13054-015-0957-y

**Published:** 2015-05-29

**Authors:** Inge K. Herrmann

**Affiliations:** Department Materials Meet Life, Swiss Federal Laboratories for Materials Science and Technology (Empa), Lerchenfeldstrasse 5, 9014 St Gallen, Switzerland

## Abstract

This viewpoint summarizes a selection of nanotechnology-based key concepts relevant to critical care medicine. It focuses on novel approaches for a trigger-dependent release of antimicrobial substances from degradable nano-sized carriers, the ultra-sensitive detection of analytes in body fluid samples by plasmonic and fluorescent nanoparticles, and the rapid removal of pathogens from whole blood using magnetic nanoparticles. The concepts presented here could significantly contribute to the prevention, diagnosis and therapy of bacterial infections in future and it is now our turn to bring them from the bench to the bedside.

Sepsis is a devastating medical condition, particularly in times when antibiotic resistance rates are rising. Despite increasing insight into the complex pathophysiology of sepsis, one of the major disappointments during the past 30 years has been the failure to convert advances in our understanding into effective new therapies [1]. Both highly specific agents and drugs with pleiotropic effects have been investigated, but with limited success. Faced with such disappointing results, the current approach to the development of sepsis therapies has been challenged by many. Translation of preclinical success stories into clinics is extremely challenging and disconnects basic sepsis research from the clinic [2]. Sepsis is not only incredibly difficult to treat, it is also hard to diagnose. Particularly in the critically ill, it is often difficult to differentiate patients urgently needing antibiotic treatment from those suffering from non-infectious systemic inflammation due to other conditions, where antibiotic treatment leads to unwanted side effects, such as *Clostridium difficile* diarrhea or other complications. Diagnostic uncertainty leads to overuse of antibiotics in hospitals and out-patient settings, and ultimately contributes to the rising rates of antimicrobial resistance, a major global healthcare challenge. Advances in intensive care along with increased awareness have helped to reduce the risk of imminent death associated with sepsis over recent decades; however, novel approaches to prevent, diagnose and treat sepsis remain major clinical needs that may only be adequately addressed by employing novel technologies.

The advent of nanotechnology has brought with it an astonishing number of novel tools enabling novel technological solutions. As the size of a system decreases, mechanical, electrical, magnetic, optical and chemical properties change when compared to macroscopic systems. While bulk gold is shiny, golden in color, inert, and conducts electricity, nano-sized gold particles with a diameter of 10 nm are red in color, catalytically active and semi-conductive [3]. Similarly, opaque materials may become transparent (bulk copper versus copper nanoparticles), stable materials may become combustible (bulk iron versus iron nanoparticles) and insoluble materials may become soluble (bulk silver versus silver nanoparticles). With the increase in surface-to-volume ratio, chemically inert materials may become catalytically active. Such dramatic changes in properties give access to a whole new world of material properties. By engineering systems at the (sub-)nanoscale, we can make use of these altered material properties and gain the ability to construct high performance systems from the bottom up. Changing the properties of materials opens up a multitude of new opportunities to improve state-of-the-art technologies; we can make systems stronger, more durable, more sensitive, multifunctional, and so on.

Here, I discuss recent developments in the field of nanoscale engineering that could potentially be employed to address major challenges in critical care medicine. I will focus on a few landmark concepts that could potentially be useful in the prevention, detection and treatment of bacterial infection and sepsis.

Generally, the best way to eliminate a problem is to eliminate its cause. Implantable devices and endotracheal tubes are a major source of hospital-acquired infection responsible for more than 40 % of all episodes of nosocomial sepsis. Silver-based compounds have been exploited for their medicinal properties for centuries; however, due to the emergence of antibiotics, the use of silver-based compounds has lost importance [4]. With the emergence of antimicrobial resistance, metallic silver in the form of silver nanoparticles has made a remarkable comeback as a potential antimicrobial agent. In contrast to older silver-containing formulations that were rapidly inactivated by the environment, nano-sized silver particles ensure a sustained release of silver ions providing antimicrobial activity [5]. However, the toxicity of silver nanoparticles remains a concern. While some studies have observed cytotoxic effects of silver nanoparticles *in vitro*, systemic toxicity of ingested silver nanoparticles is rarely seen. Supporting this, Wong and Liu [6] did not observe any overt systemic effects when injecting silver nanoparticles into mice intravenously despite the relatively high dose. Oral administration of silver nanoparticles of up to 32 ppm to healthy volunteers did not induce observable clinically important toxicity markers [7]. However, the overall significance in the *in vivo* setting and the applicability to humans remain largely unknown [6]. Remarkably, a trigger-dependent release of silver ions has been achieved by using silver nanoparticle-decorated biodegradable tricalcium phosphate particles (Fig. 1). Bacteria, when present, degrade the tricalcium phosphate matrix, thereby releasing silver that then in turn kills them. If no bacteria are present, no silver is released, hence potentially minimizing side effects when applied in clinical settings [8]. As such trigger-dependent systems greatly reduce the required dose of silver nanoparticles, they may be seen as a first step towards the safe use of silver nanoparticle-coated surfaces in clinics. Such self-sterilizing systems may significantly improve the performance of currently used silver-based coatings (for example, on surfaces and textiles), and once safety concerns have been ruled out also on catheters, endotracheal tubes, or implants [9]. Trigger-dependent silver-based antimicrobial coatings may thus become an integral part of controlling bacterial spread in hospital environments.

**Fig. 1:**
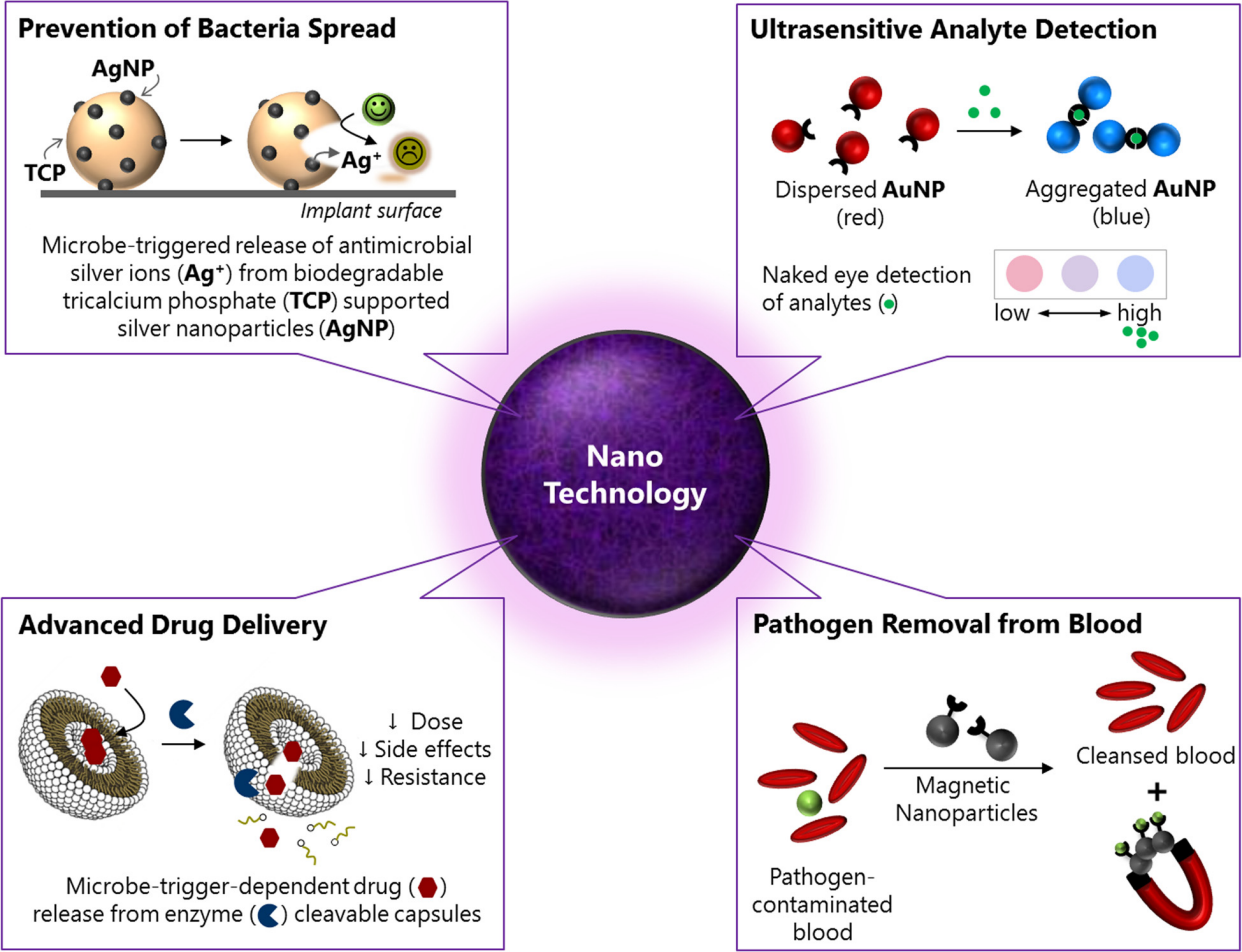
Examples of nanotechnology-based solutions that could help to prevent, detect or treat bacterial infections. Trigger-dependent release of silver ions from silver nanoparticles could help to prevent bacterial spread on surfaces of implants, cathethers or external surfaces and textiles in hospitals (top left). Aggregation of gold nanoparticles induced by the presence of an analyte changes the color of emission in a way that is visible to the naked eye, thus providing an attractive way to rapidly detect (single) biomolecules in a point-of-care compatible setting (top right). Drug molecules, such as antibiotics, can be encapsulated in enzyme-cleavable capsules. This allows the trigger-dependent and sustainable use of antibiotics (bottom left). Pathogens can be removed from whole blood by magnetic blood purification. Tiny magnetic particles capture the pathogens (for example, bacteria) and can then be separated from the blood by an external magnetic field (bottom right)

Early diagnosis is vital to ensure that early treatment is started and has a direct impact on patient outcome. Sepsis is a medical emergency and immediate treatment is required, necessitating quick, early and accurate diagnosis. However, sepsis is difficult to diagnose based solely on clinical symptoms. Blood cultures often require long incubation times, with many of them coming back as false negatives. While over 170 biomarkers have been investigated, few have entered clinical routine [10]. A singular ideal biomarker has not yet been identified and recent research efforts have focused on determining the diagnostic relevance of multiple biomarkers when used in concert [10, 11]. However, such analyses are cost-intensive and laborious as subtle changes in biomarker concentrations need to be detected within a few minutes, preferably in a point-of-care compatible setting, posing a major challenge to currently used routine measurements such as enzyme-linked immunoassays or polymerase chain reaction systems. Nanoparticle-based biosensors have the potential to improve or supersede current analytics [12]. By employing colloidal fluorescent or plasmonic nanoparticles, detection systems can be made more sensitive, reaching levels of naked-eye detection of single analytes (Fig. 1) [12]. For example, plasmonic sensors based on gold nanoparticle growth in the presence of hydrogen peroxide generated by an enzymatic reaction were employed to detect single molecules in serum by the naked eye [13, 14]. Similarly, Chapman and colleagues [15] reported on a point-of-care compatible lateral flow device enabling naked-eye detection of one nanomolar concentrations of human phospholipase in serum within 10 minutes. This assay is based on the enzyme-triggered release of multi-armed polymers from a liposome substrate that then aggregate small gold nanoparticles. The adhesion of gold nanoparticle networks to the lateral flow device then leads to the appearance of a red test line due to the localized surface plasmon resonance effect of the gold. If no enzyme (and thus no linker) is present, the gold nanoparticles flow past the test line without adhering, and thus no line is visible. Such nanoparticle-based systems enable us to detect analytes in body fluid samples at ultra-low concentrations by the naked eye in just a few minutes. Importantly, the size-dependent properties of nanoparticles (for example, gold, quantum dots) also facilitate the simultaneous detection of multiple analytes in the same system [12]. By choosing orthogonal signal generation and detection methods, a whole panel of analytes (for example, different enzymes, antigens, or nucleic acids) can be detected in a single sample at the same time [16, 17]. Such assays have the potential to decrease the time-to-result and increase sensitivity, which together with the clinical picture would allow doctors to make early clinical management decisions more confidently. Furthermore, such systems could eventually allow rapid analysis of biomarker panels at the patient’s bedside, and potentially even in resource-limited environments.

When sepsis has been diagnosed, antibiotic therapies need to be initiated. However, the treatment of sepsis patients is extremely complex and effectiveness of the antibiotic therapy heavily relies on the rapid and reliable identification of the causative microbe. In addition to diagnostic uncertainty, the pharmacokinetics of the administered antibiotics are usually altered, especially in patients with pulmonary infections, thus necessitating prolonged administration of excessive dosages, which often leads to the development of adverse side effects and antimicrobial resistance [18]. Recent studies have shown that the encapsulation of antimicrobial agents within nano-sized capsules (for example, liposomes, polymerosomes) improves pharmacokinetics and tissue distribution, target selectivity, (intracellular) delivery and overall antimicrobial effects while reducing side effects [18–20]. The properties of capsules (size, charge, fluidity) can be tailored in order to target extracellular or intracellular targets. Intracellular pathogens, such as mycobacteria, have been shown to be eradicated by using conventional liposomes that are taken up by phagocytes. Capsules with stealth properties can be designed to increase blood circulation time, achieve sustained drug release and introduce target specificity. Target specificity of liposomal formulations may be achieved either by using (positively) charged liposomes that adhere to (negatively charged) pathogens or biofilms (for example, in the lung), or by introducing specific targeting moieties, such as proteins, antibodies or oligosaccharides on the liposome surface [20]. Moreover, it has been demonstrated that antibiotics can be released in a trigger-dependent manner by using pH- or thermoresponsive capsosomes or enzyme-cleavable hyaluronic acid-based capsules [20, 21]. The hyaluronic-based capsules are cleavable by hyaluronidase secreted by bacteria, such as *Staphylococcus aureus* and *Escherichia coli*, thereby ensuring that the antibiotics are only released in the presence of bacteria (Fig. 1). In an antibiotics-independent approach, engineered liposomes have been employed as decoy targets to sequester bacterial toxins [22]. This significantly increased survival in a murine model of bacterial septicemia. The first clinical evaluations of this technique conducted on patients suffering from severe streptococcal pneumonia are scheduled for this year.

As it is not always possible to identify the causative microorganism in time, however, it is highly desirable to have treatment options that work without the need to first identify the source of infection and the causative microbe. It has recently been shown that magnetic blood purification enables rapid removal of disease-causing factors from whole blood [23–25]. In magnetic blood purification, tiny magnetic particles functionalized with capturing ligands are injected into whole blood where they bind to target compounds (Fig. 1). By applying an external magnetic gradient field, the pathogen-loaded magnetic particles can then be separated from the blood. The process can be carried out in an extracorporeal setting (similar to hemodialysis) where cleansed (pathogen-free blood) would be recirculated into the body. A multitude of compounds have been successfully removed from blood *in vitro* and *in vivo* (rat model) in recent years, including heavy metal ions [23, 26–28], small molecule drugs [23, 25], proteins (for example, cytokines) [23], endotoxins [24, 25] and bacteria [24, 25, 29]. Interestingly, by using mannose-binding lectin, Kang and colleagues [25] demonstrated >90 % removal of bacteria from blood without the need for identification of the bacteria prior to treatment in a rodent model. This approach could effectively bridge the gap until the causative microbes have been identified (for example, by blood culture or by analyzing the pathogens bound to the magnetic beads) and optimal antibiotic therapy can be initiated. The group is currently testing the biospleen device in a pig model and it is expected that the system will enter clinical evaluation in a couple of years.

As for every new technology, safety evaluations are a pivotal and most often rate-determining step in the translational process. Toxicity, hemocompatibility, off-target effects as well as long-term effects of nanomaterials need to be evaluated case by case (and under relevant conditions) before a nanotechnology-enabled therapy can be considered safe. Classical toxicology protocols may not always be suitable for the assessment of nanomaterials as their interaction with biological systems is governed by a series of characteristics not found for molecules: chemically reactive interfaces, the combination of solid-state properties and mobility, and altered biodistribution (more details can be found in [30]). In order to minimize adverse side-effects, therapeutic applications should focus on biodegradable nanomaterials consisting of non-toxic elements. Ultimately, successful translation of nanotechnology-enabled concepts heavily relies on the close collaboration between scientists and clinicians in interdisciplinary teams. Taken together, nanotechnology-based approaches offer a multitude of opportunities and immense potential for improved detection and treatment of bacterial infections and beyond. It is now our turn to take these technologies and bring them from the lab to the bedside by making safe use of them.

## References

[1] Angus DC, van der Poll T (2013). Severe sepsis and septic shock. N Engl J Med.

[2] Rittirsch D, Hoesel LM, Ward PA (2007). The disconnect between animal models of sepsis and human sepsis. J Leukocyte Biol.

[3] Kelly KL, Coronado E, Zhao LL, Schatz GC (2003). The optical properties of metal nanoparticles: the influence of size, shape, and dielectric environment. J Phys Chem B.

[4] Atiyeh BS, Costagliola M, Hayek SN, Dibo SA (2007). Effect of silver on burn wound infection control and healing: review of the literature. Burns.

[5] Rai M, Yadav A, Gade A (2009). Silver nanoparticles as a new generation of antimicrobials. Biotechnol Adv.

[6] Wong KKY, Liu X (2010). Silver nanoparticles-the real “silver bullet” in clinical medicine?. Med Chem Comm.

[7] Munger MA, Radwanski P, Hadlock GC, Stoddard G, Shaaban A, Falconer J (2014). In vivo human time-exposure study of orally dosed commercial silver nanoparticles. Nanomedicine.

[8] Loher S, Schneider OD, Maienfisch T, Bokorny S, Stark WJ (2008). Micro-organism-triggered release of silver nanoparticles from biodegradable oxide carriers allows preparation of self-sterilizing polymer surfaces. Small.

[9] Kollef MH, Afessa B, Anzueto A, Veremakis C, Kerr KM, Margolis BD (2008). Silver-coated endotracheal tubes and incidence of ventilator-associated pneumonia: the nascent randomized trial. JAMA.

[10] Pierrakos C, Vincent J-L (2010). Sepsis biomarkers: a review. Crit Care.

[11] Vincent J-L, Teixeira L (2014). Sepsis biomarkers. Value and limitations. Am J Respir Crit Care Med.

[12] Howes PD, Chandrawati R, Stevens MM (2014). Colloidal nanoparticles as advanced biological sensors. Science.

[13] Wang S, Bi S, Wang Z, Xia J, Zhang F, Yang M (2015). A plasmonic aptasensor for ultrasensitive detection of thrombin via arrested rolling circle amplification. Chem Commun.

[14] de la Rica R, Stevens MM (2012). Plasmonic ELISA for the ultrasensitive detection of disease biomarkers with the naked eye. Nat Nanotechnol.

[15] Chapman R, Lin Y, Burnapp M, Bentham A, Hillier D, Zabron A (2015). Multivalent nanoparticle networks enable point-of-care detection of human phospholipase-A2 in serum. ACS Nano.

[16] Geißler D, Charbonnière LJ, Ziessel RF, Butlin NG, Löhmannsröben H-G, Hildebrandt N (2010). Quantum dot biosensors for ultrasensitive multiplexed diagnostics. Angew Chem Int Ed Engl.

[17] Lowe SB, Dick JAG, Cohen BE, Stevens MM (2012). Multiplex sensing of protease and kinase enzyme activity via orthogonal coupling of quantum dot-peptide conjugates. ACS Nano.

[18] Omri A, Suntres ZE, Shek PN (2002). Enhanced activity of liposomal polymyxin B against Pseudomonas aeruginosa in a rat model of lung infection. Biochem Pharmacol.

[19] Walsh TJ, Finberg RW, Arndt C, Hiemenz J, Schwartz C, Bodensteiner D (1999). Liposomal amphotericin B for empirical therapy in patients with persistent fever and neutropenia. N Engl J Med.

[20] Drulis-Kawa Z, Dorotkiewicz-Jach A (2010). Liposomes as delivery systems for antibiotics. Int J Pharmaceutics.

[21] Baier G, Cavallaro A, Vasilev K, Mailänder V, Musyanovych A, Landfester K (2013). Enzyme responsive hyaluronic acid nanocapsules containing polyhexanide and their exposure to bacteria to prevent infection. Biomacromolecules.

[22] Henry BD, Neill DR, Becker KA, Gore S, Bricio-Moreno L, Ziobro R (2015). Engineered liposomes sequester bacterial exotoxins and protect from severe invasive infections in mice. Nat Biotechnol.

[23] Herrmann IK, Urner M, Koehler FM, Hasler M, Roth-Z’Graggen B, Grass RN (2010). Blood purification using functionalized core/shell nanomagnets. Small.

[24] Herrmann IK, Urner M, Graf S, Schumacher CM, Roth-Z’graggen B, Hasler M (2013). Endotoxin removal by magnetic separation-based blood purification. Adv Healthcare Mater.

[25] Kang JH, Super M, Yung CW, Cooper RM, Domansky K, Graveline AR (2014). An extracorporeal blood-cleansing device for sepsis therapy. Nat Med.

[26] Lee HY, Bae DR, Park JC, Song H, Han WS, Jung JH (2009). A selective fluoroionophore based on BODIPY-functionalized magnetic silica nanoparticles: removal of Pb2+ from human blood. Angew Chem Int Ed Engl.

[27] Wang L, Yang Z, Gao J, Xu K, Gu H, Zhang B (2006). A biocompatible method of decorporation: bisphosphonate-modified magnetite nanoparticles to remove uranyl ions from blood. J Am Chem Soc.

[28] Herrmann IK, Schlegel A, Graf R, Schumacher CM, Senn N, Hasler M (2013). Nanomagnet-based removal of lead and digoxin from living rats. Nanoscale.

[29] Lee J-J, Jeong KJ, Hashimoto M, Kwon AH, Rwei A, Shankarappa SA (2014). Synthetic ligand-coated magnetic nanoparticles for microfluidic bacterial separation from blood. Nano Lett.

[30] Stark WJ (2011). Nanoparticles in biological systems. Angew Chem Int Ed Engl.

